# Switching the poles in sexual and reproductive health research: implementing a research capacity-strengthening network in West and North Africa

**DOI:** 10.1186/s12978-016-0203-3

**Published:** 2016-08-08

**Authors:** Jean-Paul Dossou, Bouchra Assarag, Alexandre Delamou, Karen Van der Veken, Loubna Belaid, Moctar Ouédraogo, Sonia Khalfallah, Hayet Aouras, Mohamed Diadhiou, Raïmi Fassassi, Thérèse Delvaux

**Affiliations:** 1 Centre de Recherche en Reproduction Humaine et en Démographie, CNHU/HKM, Avenue Jean-Paul, Cotonou, Benin; 2 Department of Public Health, Institute of Tropical Medicine, 155 Nationalstraat, 2000 Antwerp, Belgium; 3 Ecole Nationale de Santé Publique, Rue Lamfadel Cherkaoui, Madinat Al Irfane, Rabat, Morocco; 4 Centre National de Formation et de Recherche en Santé Rurale de Mafèrinyah, Forécariah, Guinée; 5 Woman and Child Health Research Centre, Institute of Tropical Medicine, 155 Nationalstraat, 2000 Antwerp, Belgium; 6 École de Santé Publique, Université de Montréal, 7101 Av du Parc H3N1X9, Montréal, Québec Canada; 7 Agence de Formation, de Recherche et d’Expertise en Santé pour l’Afrique, 773, Rue Guillaume Ouédraogo, Bobo-Dioulasso, Burkina Faso; 8 Direction Régionale de la Santé de Nabeul, Rue de la Révolution, Nabeul, Tunisie; 9 Registre des Cancers d’Annaba, Université d’Annaba, CHU, Annaba, Algérie; 10 Centre Régional de Formation, de Recherche et de Plaidoyer en Santé de la Reproduction, Maternité Hôpital Aristide Le Dantec, Avenue Pasteur, Dakar, Sénégal; 11 Ecole Nationale Supérieure de Statistique et d’Economie Appliquée, Boulevard Mitterrand, Cocody, Abidjan, Côte d’Ivoire

**Keywords:** Sexual and reproductive health, Research capacity, Africa, Networking, Implementation, Collective intelligence, Switching the poles

## Abstract

Health research capacities have been improved in Africa but still remain weak as compared to other regions of the World. To strengthen these research capacities, international collaboration and networking for knowledge and capacity transfer are needed. In this commentary, we present the Network for Scientific Support in the field of Sexual and Reproductive Health in West and North Africa, its priority research topics and discuss its implementation process. Established in January 2014, the Network aims at generating human rights and gender-based research fully carried out and driven by South based institutions. It is composed of 12 institutions including the Institute of Tropical Medicine of Antwerp (Belgium) and 11 institutions from eight Francophone West and North African countries. The key areas of interest of this network are health policies analysis and health system research in family planning, HIV prevention among vulnerable groups, quality of care and breast cancers. Since it started, seventeen research proposals based on locally relevant research questions have been developed. Among the seventeen proposals, eleven have been implemented. Several research institutions enhanced linkages with local representations of international partners such as UNFPA. The network is committed to strengthening methodological research capacities and soft skills such as fundraising, advocacy and leadership. Such competencies are strongly needed for developing an effective South-based leadership in Sexual and Reproductive Health research, and for achieving the Sustainable Development Goals.

## Résumé

En Afrique, les capacités de recherche en santé s’améliorent, mais elles restent faibles comparativement aux autres régions du monde. Pour renforcer ces capacités, la collaboration internationale et la mise en réseau des institutions pour améliorer le transfert des connaissances et des capacités sont nécessaires. Dans ce commentaire, nous présentons l’expérience du Réseau d’Appui Scientifique en Santé Sexuelle et Reproductive en Afrique du Nord et de l’Ouest. Nous décrivons et discutons le processus de sa mise en œuvre. Le réseau a été lancé en Janvier 2014 et son objectif principal est de générer des travaux de recherche basés sur les droits humains et le genre, initiés et entièrement conduits par des institutions basées en Afrique. Les principaux domaines d’intérêt sont les politiques et les systèmes de santé en matière de planification familiale, la prévention du VIH au sein des populations vulnérables, la qualité des soins et la réduction du fardeau lié aux cancers du sein. Le réseau est composé de 12 institutions basées dans huit pays francophones en Afrique de l’Ouest et du Nord, et de l’Institut de Médecine Tropicale d’Anvers (Belgique). Depuis lors, dix-sept propositions de recherche basées sur des questions pertinentes au niveau local ont été développées. Parmi ces propositions, onze ont été mises en œuvre. Plusieurs institutions de recherche ont initié ou renforcé des collaborations scientifiques avec les représentations locales des partenaires internationaux tels que le FNUAP. Le réseau est déterminé à renforcer ses capacités en matière en recherche ainsi que ses compétences génériques telles que la recherche de financement, le plaidoyer et le leadership. Ces compétences sont essentielles pour le développement d’un leadership en recherche basé en Afrique, capable de soutenir la réalisation des Objectifs de Développement Durable.

## Background

Health research capacities in Africa are improving in several domains [1]. In fact, the overall number of health scientific publications attributable to the WHO African Region has substantially grown from 3,623 in 2000 to 12,709 in 2014, with a research productivity increasing significantly by 10.3 % per year [2].

Despite this growing trend, the research productivity in Africa is still weak as compared to other regions of the World. In biomedical research for instance, there were 3.5 publications per million population per year attributable to Africa between 1990 and 2000 compared to 136.9 and 341.3 respectively in Europe and in North America [3]. In addition, research projects conducted in Africa are usually built and led by researchers from high-income countries, with African researchers playing an ancillary role [4].

Within Africa, the research capacity is unequally distributed. South Africa and North African countries such as Egypt detain a record of the best academic institutions on the continent since decades. Anglophone countries are as well more productive as compared to francophone ones. From 2000 to 2014, three Anglophone countries (South Africa, Nigeria and Kenya) hold 52 % of the overall absolute number of publications of the WHO African region [2]. Senegal is the first francophone country ranking at the 9^th^ level in the top ten publishing countries in the WHO African region [2], considering the number of publications normalized by the population size.

Nevertheless, a strong research capacity all over the African continent is an important condition to achieve the Sustainable Development Goals (SDGs). SDGs emphasize sustainability, resilience and equity. Achieving those goals needs scientific efforts for measuring the burden of existing or emerging health problems, evaluating existing interventions, (re) designing and implementing bundle of context sensitive and adaptive policies [5]. The success of this process requires strong national system for health research capable of successfully managing the whole cycle of research agendas, from formulating research questions that are relevant to local needs to the effective use of research findings for decision-making and action.

Strengthening these research capacities requires more international collaboration and networking for knowledge and capacity transfer [4, 6]. There is a need for an actual shift from the top-down foreign research-for-aid approach to a bottom-up process as expressed in the Paris declaration since 2005 [7].

In the field of Sexual and Reproductive Health (SRH), existing networks mainly include Anglophone countries [4]. The Network for Scientific Support in the field of SRH (NetSRH) in West and North Africa was created in 2014 within the switching the poles capacity building program of the Institute of Tropical Medicine of Antwerp (ITM) to fill this gap by focusing on francophone countries. In this commentary, we present the network, its priority research topics and discuss the implementation process and achievements so far.

### Implementation process of the SRH research network

The NetSRH aims at developing and strengthening scientific capacities and collaborations between institutions that conduct research in SRH that can be translated into policy and practice. The idea of creating a network that promotes a human rights and gender based approach in SRH research in the South emerged from discussions among staff members, partners and alumni members of the ITM. Preliminary consultations with stakeholders in eligible institutions followed, for expectations analysis and goals alignment. This process led to the involvement of 11 institutions from West (Benin, Burkina Faso, Guinea, Ivory coast, Senegal) and North (Algeria, Morocco, Tunisia) Africa, with the ITM. The focus on francophone countries aimed at contributing to the reduction of the French-English gap in terms of research capacity within the continent. NetSRH also represents an opportunity to connect with similar networks in Anglophone countries, and seeks to promote a greater South-South collaboration. The NetSRH adopted a typical network governance model with a multi-centric system of interaction between interdependent actors, negotiation rationality for decision making, and insurance of compliance through trust and political obligation [8].

## Results - early achievements

Since the launching of the network in January 2014, tree workshops have been organized successfully. A short network meeting was also organized as a side event to the ITM/*Ecole Nationale de Santé Publique,* Rabat, Morocco (ENSP) colloquium on maternal and neonatal health in November 2015. ENSP hosted the kick–off workshop in February 2014 that offered venue for methodological training and definition of priority research topics. Participants at this meeting were updated on human rights and gender based research approach, and research methodology by both experts from ITM and the south institutions. A number of issues were addressed such as how to conduct a systematic literature review, writing a proposal, qualitative research methods, and ethics in research. A collective research priority setting session during this meeting identified operational, health policy and system research in family planning, HIV prevention among vulnerable groups, quality of care at delivery and breast cancers, as key research priorities.

The network launched a blog as a tool to promote an internal communication and cohesion between member institutions. The blog is moderated by experts from south institutions, to improve ownership and set the stage for the development of political soft skills and leadership capacities within the network.

Members wrote seventeen research proposals, based on research questions they perceived as locally relevant. Of these, fourteen proposals underwent several rounds of peer review, online and face-to-face during the second meeting organized by the *Centre Régional de Formation, de Recherche et de Plaidoyer en Santé de la Reproduction* in Dakar, Senegal, in January 2015. South institutions were paired in the review of their respective proposals. Each institution received personalized written comments on the format, the structure and the content of its proposal, including from the ITM. South institutions discussed those comments with each other, and then each institution presented a summary of the useful inputs and suggestions received in a plenary session. Fifteen proposals were submitted locally to at least one funding institution. As of February 2016, this process led to eleven researches directly or indirectly initiated via the network that are being conducted. Progress has been made in the capacities of south institutions in engaging and negotiating with national offices of international agencies for funding. In Morocco for instance, the ENSP was successful in getting its protocols on “underutilization of the intrauterine devices” and “violence against women and quality of care to mothers and newborns” funded respectively by UNFPA and WHO EMRO. Although the first proposals proposed by the *Centre de Recherche en Reproduction Humaine et en Démographie* (CERRHUD) in Benin were not funded, the research centre was strengthened via the network to submit two other research proposals for funding to the local office of the UNFPA in Cotonou, Benin. As a result of this contact, an action research developed by CERRHUD on implementing a Maternal Death Surveillance and Response system in two health districts in Benin has been funded by UNFPA Country office.

Beside this North–south and South-South capacity transfer, there is a South–north transfer of capacities and knowledge in describing and analyzing local and contextual evidences. For instance, critical local contextual evidences have been shared with ITM, about contraceptive seeking behaviors in Benin, in a collective effort to design a research proposal that has been submitted to the Gate Foundation in March 2016.

Within the network, beyond national studies, a clear commitment is emerging for designing and implementing multi-countries research. It results from the dynamics groups that is building up along planned multilateral as well as emerging bilateral interactions between participating institutions. Even if, so far no budget is available for conducting joint research, this strategy is part of a set of initiatives, including the full implementation of the network governance, to support the sustainability of the network beyond the current funding. Indeed, at this early phase the ITM is accountable vis-à-vis the funder for the project management and therefore holds a higher steering position as compared to the other institutions. But this is clearly identified as a temporary situation that the network is committed to overcome as quickly as possible, by establishing a coordinating committee, in which all the South institutions will play a greater role. Other initiatives for sustainability include: i) the use of free online platforms and tools (Google group, webinars, online shared repositories) to create a cheap, permanent and institutionalized channels of interaction between all the institutions; ii) the design of multi-country research proposals based on common research interests to provide opportunities for peer training and face to face meetings.

## Discussion

The NetSRH aims at strengthening research capacity by networking and collaboration as do several other networks in SRH [4] or in other fields [9]. The implementation process emphasizes the collective and bottom-up approach in setting up research priorities and generating research questions. By supporting south institutions in developing their own protocols instead of merely implementing proposals developed by international agencies or North institutions, the NetSRH clearly focuses not only on the development of the methodological capacities but also on the shift of power and responsibilities. In its capacity building program, the NetSRH emphasizes advocacy and negotiation with national agencies for fund-raising instead of merely providing external financial resources to finance the proposal developed. It also promotes soft political skills such as scientific communication during international events, facilitation of online forums, provision of constructive feedbacks in peer reviews processes etc. This demonstrates at least the commitment of the network to lead to an effective ‘switching the poles’ in research. This overall implementation approach has the potential not only to lead to studies developed and fully carried out by South institutions, but also to improve credibility and visibility of individual researchers and institutions members of the network at national, regional and international levels.

It is clear that achieving the overall goal of health research capacity strengthening requires a bundle of interventions [6, 9] more than soft and methodological capacities transfer. For instance developing competitive and structuring grant and fellowship schemes administered by African institutions and providing institutional support for infrastructure, management, technical services and strategic development planning are required. There is a need for improving research environment and supporting individuals to engage talented researchers, to sustain their commitment and to promote their lighting up [9]. This involves a national political commitment in building a set of “people, institutions, and activities whose primary purpose in relation to research is to generate high-quality knowledge that can be used to promote, restore, and/or maintain the health status of populations” [6]. Such a system for health research needs an oversight governance (stewardship), an adequate financing, the creation and sustaining of resources, the production and utilization of research findings [6].

All those dimensions are beyond the scope of the NetSRH only in a short run. However, the network can take better profit of the momentum on health policy and system research strengthening and of new technologies of information and communication, to optimize the fulfillment of its potential. Indeed, there is still an important room for progression to consider that NetSRH is fully implemented as a social network: a set of social actors, sets of dyadic ties, and other social interactions between them [10]. Even if the set of institutions is built and some formal and informal interactions start happening, we are still far of triggering the mechanism of a collective intelligence within the network [11].

Such a network should have a clear added value for each member, a clear benefit impossible in absence of the network. Realizing this added value is the optimal outcome of the implementation process of this singular network that took clearly the stance of surviving on its own without a long-term dependency on any external funder.

## Conclusion

Strengthening health research system in Africa is a key for an effective implementation of SDGs strategies. The NetSRH has identified priority research questions that require operational, health policy and system research. The NetSRH is working to induce a strong ownership and a group dynamic capable of maintaining an optimal exchange and collaboration, mobilizing internal and external resources for its effectiveness and sustainability. There is a strong commitment toward those ends with perspectives for developing the existing network ties into a growing south-south and Anglo-French collaboration.

## Abbreviations

CERRHUD, Centre de Recherche en Reproduction Humaine et en Démographie; ENSP, Ecole Nationale de Santé Publique of Rabat, Morocco; ITM, Institute of Tropical Medicine of Antwerp, Belgium; NetSRH, network for scientific support in the field of sexual and reproductive health; SRH, sexual and reproductive health; UNFPA, United Nations Population Fund; WHO EMRO: Word Health Organization Eastern Mediterranean Regional Office
